# Association of polymorphism in genes encoding κB inhibitors (IκB) with susceptibility to and phenotype of Graves' disease: a case-control study

**DOI:** 10.1186/1756-6614-2-10

**Published:** 2009-11-03

**Authors:** Alina Kurylowicz, Piotr Miśkiewicz, Ewa Bar-Andziak, Janusz Nauman, Tomasz Bednarczuk

**Affiliations:** 1 Department of Endocrinology, Mossakowski Medical Research Center, Polish Academy of Science, Pawinskiego 5, 02-106 Warsaw, Poland; 2 Department of Endocrinology, Medical University of Warsaw, Banacha 1A, 02-097 Warsaw, Poland

## Abstract

**Background:**

Genes related to the nuclear factor-κB (NF-κB), a key transcription factor involved in regulation of immune responses, are interesting candidates for association studies in autoimmune disorders. The aim of this study was to investigate an association of polymorphisms in two genes encoding NF-κB inhibitors: *IKBL *(encoding inhibitor of κB-like) and *NFKBIA *(encoding κB inhibitor α), withsusceptibility to and phenotype of Graves' disease (GD).

**Methods:**

A population-based, case-control association study comprising 481 patients with GD and 455 healthy controls was performed. We analyzed 3 single nucleotide polymorphisms (SNPs) in *IKBL *[promoter region -62T/A substitution (rs2071592), intron 1 C/T substitution (rs2071591) and exon 4 T/C substitution (rs3130062)] and 3 SNPs in *NFKBIA *[G/A substitution in 3' untranslated region (rs696) and two promoter region polymorphisms -297C/T (rs2233409) and -826C/T (rs2233406)] by the PCR-restriction fragment length polymorphism (RFLP) method.

**Results:**

The two SNPs in *IKBL *(rs2071592 and rs2071591) were in a strong linkage disequilibrium (D' = 0.835) and the AT haplotype was associated with susceptibility to GD (p < 10^-4^, OR = 1.61 [95%CI:1.21-2.14]). Moreover subgroup analysis revealed a gen-gen interaction between the investigated *IKBL *haplotype and HLA-DRB1*03 allele (p < 10^-4^). The investigated *NFKBIA *SNPs were not associated with susceptibility to GD. However, when correlated with phenotype, the -297T (rs2233409) and -826T (rs2233406) alleles were associated with the development of clinically evident ophthalmophaty (p = 0.004, p_c _= 0.07, OR = 1.65 [95%CI: 1.18-2.38] and p = 0.002, p_c _= 0.036, OR = 1.67 [95%CI: 1.20-2.36], respectively).

**Conclusion:**

Our results suggest that SNPs in genes encoding NF-κB inhibitors may contribute to the development and clinical phenotype of GD.

## Background

The nuclear factor-κB (NF-κB) is an ubiquitous transcription factor of particular importance in normal inflammatory and immune responses and there is a growing amount of evidence that its deregulated activation may play a key role during development of common inflammatory and autoimmune diseases [1].

In mammals the NF-κB family consists of two types of proteins: the first group includes p105 and p100 that after proteolysis generate short active molecules (p50 and p52, respectively); whereas the second group includes p65, c-Rel and RelB proteins, that posses transcriptional activations domains. Both groups are characterized by the presence of a Rel homology domain which contains a nuclear localization sequence (NLS) and is involved in DNA binding. In non-stimulated cells the NF-κB proteins form homo- or heterodimers that are sequestered in cytoplasm *via *interaction with κB inhibitors (IκB). The IκB family comprises several members, including IκBα, IκBβ, IκBε and other related proteins (such as inhibitor of κB-like - IκBL or Bcl-3) that interact with NLS in the NF-κB proteins and in this way prevent their nuclear translocation. Numerous activating signals can trigger transduction pathways leading to dissociation of NF-κB from IκBs. The first step of this process involves activation of IκB kinases (IKK). IKK phosphorylate serine residuals in the N-terminal part of the IκB proteins that creates a binding site for subunits of the ubiquitin ligase complex and results in rapid polyubiquitination of IκB followed by its proteasomal degradation. Dissociation of IκB exposes the NLS in NF-κB proteins leading to their nuclear translocation and binding to promoters of target genes [2].

Therefore the coordinated degradation and resynthesis of the IκB proteins regulates NF-κB activity and any change in this delicate balance can interfere with normal NF-κB functions [3]. Given the important role of the NF-κB transduction pathway in immune responses, NF-κB related genes are interesting candidates for susceptibility genes in autoimmune disorders. In our previous study we found that a promoter polymorphism (-94ins/del ATTG) in the gene encoding p105/p50, may be associated with susceptibility to and/or phenotype of Graves' disease (GD) [4]. Polymorphism within genes encoding IκB proteins were found to be associated with development of other autoimmune conditions including: multiple sclerosis, rheumatoid arthritis, type 1 diabetes mellitus, systemic lupus erythematosus and Crohn's disease in different populations [5-12].

In the present study first, we analyzed an association of selected, potentially functional, polymorphisms in two genes encoding κB inhibitors: *IKBL *encoding IκBL and *NFKBIA *encoding IκBα with susceptibility to GD in Polish Caucasians. The analyzed *IKBL *and *NFKBIA *single nucleotide polymorphisms (SNPs) were selected based on previous associations studies in other autoimmune diseases. Next, we searched for a correlation with clinical phenotype of GD (gender, age of disease onset, presence of ophthalmophaty and family history of autoimmune thyroid diseases - AITD). Finally, we analyzed gene-gene interactions between IκB alleles and other established GD susceptibility markers (HLA-DRB1*03, *CTLA-4 *49A/G and *PTPN22 *1858C/T).

## Subjects and methods

### Subjects

Patients with GD (N = 481) were consecutively recruited from the Department of Endocrinology, Medical University of Warsaw, as described previously [13]. All individuals were of Caucasian origin and their clinical characteristics are shown in Table 1. The diagnosis of GD was based on clinical and biochemical symptoms of hyperthyroidism and was confirmed by the presence of diffuse goiter, detectable TSH receptor autoantibodies (TRAK Lumitest, B.R.A.H.M.S Diagnostica GmbH, Germany) and/or increased radioiodine uptake. The severity of ophtalmopathy was assessed according to the NOSPECS classification. Patients with proptosis (NOSPECS class III), extraocular muscle dysfunction (class IV), exposure keratitis (class V) and optic neuropathy (class VI) were considered clinically evident.

**Table 1:** Clinical characteristics of patients with Graves' disease (GD).

**Characteristics**	**N**	**(%)**
Male	481	97 (20.2)
Age of onset of GD (yr)*	448	38.79 ± 14.6
Disease duration (yr)*	447	4.72 ± 6.26
Ophthalmopathy (NOSPECS class III and higher)	459	170 (37.0)
Cigarette smokers	435	192 (44.1)
Family history of AITD	422	133 (31.5)
Therapy for hyperthyroidism:	457	
Antithyroid drugs		150 (32.9)
Radioactive iodine		230 (50.3)
Surgery		77 (16.8)

N - number of patients available for analysis.

* Age of onset and disease duration are presented as mean ± SD.

AITD - autoimmune thyroid diseases.

The control group comprised 455 ethnically matched healthy subjects (119 male, 336 female, mean age 30.47 ( ± 10.07 years)) who had no family history of autoimmune diseases. The research program was approved by the Local Ethical Committees, and written informed consent was obtained from all of the participants.

### Genotyping

Genomic DNA was extracted from peripheral blood mononuclear cells by the salting-out method [14]. Genotyping of the selected polymorphisms in the *IKBL *and *NFKBIA *genes was performed by PCR amplification followed by digestion with a proper restriction enzyme (restriction fragment length polymorphism method - RFLP) [see Additional files 1]. The obtained restriction fragments were visualized on a 3% agarose gel. In the *IKBL *gene three polymorphisms were analyzed: a T/A substitution in position -62 of the promoter (rs2071592), a C/T substitution in intron 1 (rs2071591) and a T/C substitution in exon 4 (rs3130062), whereas in the *NFKBIA *gene: a G/A substitution in 3' untranslated region (3'UTR, rs696) and two promoter polymorphisms: a C/T substitution in position -297 (rs2233409) and C/T substitution in position -826 (rs2233406) were studied.

Genotyping of the *HLA-DRB1*, *CTLA-4 *and *PTPN22 *polymorphisms was performed as reported previously [13,15,16].

### Statistical analysis

Genotype frequencies were compared between groups by chi-square (χ^2^) test on 2 × 2 or 3 × 2 contingency tables using Statistica software package (StatSoft Inc., Tulsa, OK) and p values less than 0.05 were considered significant. Additionally Bonferoni's correction for multiple testing was applied and a corrected p value (p_c_) is also presented. Odds ratios (OR) with 95% confidence intervals were calculated by the method of Woolf [17]. Linkage disequilibrium (LD) was analyzed using the pairwise LD measure D' and haplotype blocks were constructed from population genotype data with the use of Haploview software [18], using the default algorithm for generating haplotype blocks based on methods established by Gabriel *et al. *[19]. A D' value of 1 indicates complete LD between the two markers, D' value greater than 0.8 - strong LD, 0.2-0.8 - incomplete LD, whereas a D' less than 0.2 - negligible LD [20]. Power analysis was performed using the DSS software available online .

## Results

### Genotyping results, linkage disequilibrium analysis and construction of haplotypes

Frequencies of *IKBL *and *NFKBIA *genotypes observed in patients with GD and in healthy subjects are shown in Table 2. All cases and controls in the studied cohorts were in Hardy-Weinberg equilibrium.

**Table 2:** Distribution of *IKBL *and *NFKBIA *genotypes in patients with Graves' disease (GD) and in healthy controls

**Gene**	**polymorphism**	**genotype**	**GD (%)N = 481**		**Controls (%)N = 455**		**p/p_c _value OR (95%CI)**
*IKBL*	promoter -62	TT	183	(38.0)	226	(49.7)	p = 0.001*
		AT	221	(46.0)	181	(39.8)	p_c _= 0.018
		AA	77	(16.0)	48	(10.5)	OR = 1.54 (1.20-1.99)
	
	intron 1	CC	186	(38.7)	223	(49.0)	p = 0.001*
		CT	240	(49.9)	187	(41.1)	p_c _= 0.018
		TT	55	(11.4)	45	(9.9)	OR = 1.52(1.18-1.98)
	
	exon 4	TT	441	(91.7)	414	(91.0)	NS
		CT	40	(8.3)	41	(9.0)	
		CC	0	(0.0)	0	(0.0)	

*NFKBIA*	3'UTR	GG	160	(33.2)	134	(29.5)	NS
		AG	235	(48.9)	234	(51.4)	
		AA	86	(17.9)	87	(19.1)	
	
	promoter -297	CC	333	(69.2)	310	(68.1)	NS
		CT	134	(27.9)	132	(29.0)	
		TT	14	(2.9)	13	(2.9)	
	
	promoter -826	CC	318	(66.1)	291	(64.0)	NS
		CT	147	(30.6)	151	(33.2)	
		TT	16	(3.3)	13	(2.8)	

* p value was calculated by a by chi-square (χ^2^) test using a 2 × 2 table to compare the frequency of genotypes possessing the A allele *vs*. TT homozygotes and corrected (p_c>_) for the number of tests performed.

** p value was calculated by a by chi-square (χ^2^) test using a 2 × 2 table to compare the frequency of genotypes possessing the T allele *vs. *CC homozygotes and corrected (p_c_) for the number of tests performed.

NS - non significant.

The frequency of the allele -62A in *IKBL *was greater in GD compared to controls (39.0% *vs. *30.4%, p = 0.0001, p_c _= 0.0018; OR = 1.46 [95% CI:1.20-1.77]). A significant association was also observed for genotypes (p = 0.001, p_c _= 0.018): genotypes possessing the A allele (AA homozygotes and AT heterozygotes) were found more frequently in GD patients (62.0% *vs. *50.3%, p = 0.001, p_c _= 0.018) producing OR of 1.54 [95% CI:1.20-1.99] suggesting a dominant model of inheritance (statistical power - SP = 95.1%). For the intronic C/T substitution, an association of the T allele with GD was found (36.1% *vs. *30.4%, p = 0.01, p_c _= 0.18; OR = 1.29 [95% CI:1.06-1.56]) and consistently, genotypes possessing this allele (TT homozygotes and CT heterozygotes) were more frequent in GD patients (61.3% *vs. *51.0%, p = 0.001, p_c _= 0.018; OR = 1.52 [95% CI:1.18-1.98], SP = 88.9%). The T/C SNP in exon 4 of the *IKBL *was not associated with GD.

A strong LD between the *IKBL *promoter -62T/A and intron 1 C/T polymorphisms was observed (D' = 0.835). Subsequently, the distribution of *IKBL *haplotypes differed significantly between GD patients and healthy subjects (p < 10^-4^, Table 3), with an increased frequency of the AT haplotype in GD patients (34.6% *vs. *24.6%, OR = 1.61 [95%CI: 1.21-2.14]).

**Table 3:** Frequencies of *IKBL *haplotypes in patients with Graves' disease (GD) and in healthy controls.

**Gene**	**polymorphism 1**	**polymorphism 2**	**GD** **N = 481(%)**	**Controls** **N = 455 (%)**
***IKBL***	**promoter -62 T/A**	**intron 1 C/T**		

haplotypes	T	C	287 (59.7)	299 (65.6)
	A	T	167 (34.6)	112 (24.6)
	A	C	20 (4.2)	27 (6.0)
	T	T	7 (1.5)	17 (3.8)

Haploview software was used to construct haplotypes from population genotype data and to assess possible difference in their distribution between GD vs. healthy subjects (using a nonparametric test). The distribution of *IKBL *haplotypes differed significantly between GD patients and healthy subjects (p < 10^-4^).

None of the three SNPs analyzed in the *NFKBIA *gene was found to be associated with GD (Table 2).

### Correlation with the clinical phenotype of GD

Distribution of *IKBL *and *NFKBIA *genotypes was compared in subgroups of GD patients stratified by clinical parameters, including gender, age of GD onset, presence of clinically evident ophthalmopathy, family history of autoimmune thyroid diseases and smoking habits.

In case of two promoter polymorphisms studied in the *NFKBIA *gene an association with the development of clinically evident ophthalmopathy was detected, however, after Bonferroni adjustment, some results did not remain significant (Table 4). For the -297C/T SNP, the allele T (21.2%*vs. *13.8%, p = 0.004; p_c _= 0.072; OR = 1.67 [95%CI: 1.18-2.38]), as well as genotypes inclusive this variant (TT homozygotes and CT heterozygotes; 37.1% *vs. *26.3%, p = 0.01; p_c _= 0.18; OR = 1.65 [95%CI: 1.10-2.48], SP = 67.8%), were observed more frequently in the subgroup of patients with clinically evident ophthalmopathy. Similarly, for the -826C/T polymorphism, an association of the allele T (and genotypes possessing this allele) with the presence of ophthalmopathy was detected (23.2% *vs. *15.2%, p = 0.002; p_c _= 0.036; OR = 1.68 [95%CI: 1.20-2.36] for alleles and 40.6% *vs. *29.1%, p = 0.01; p_c _= 0.18; OR = 1.67 [95%CI: 1.12-2.48] for genotypes, SP = 71.1%). Since these two polymorphisms were in a strong LD, analysis of haplotypes was also performed (Table 5). The distribution of *NFKBIA *promoter haplotypes differed significantly between subgroups of GD subjects stratified by the presence of clinically evident ophthalmopathy (p = 0.003), with an increased frequency of the T•T haplotype in patients with NOSPECS ≥ III (18.4% *vs. *11.3%, OR = 1.73 [95%CI: 1.02-2.94]).

**Table 4:** Distribution of *IKBL *and *NFKBIA *genotypes in subgroups of patients with Graves' disease stratified by clinical activity of thyroid associated ophtalmopathy assessed according to the OSPECS classification.

**Gene**	**polymorphism**	**genotype**	**Ophtalmopathy**				**p/p_c _value OR (95%CI)**
				
			**NOSPECS ≥ III (%)** **N = 170**		**NOSPECS ≤ II (%)** **N = 289**		
*IKBL*	promoter -62	TT	65	(38.2)	105	(36.3)	NS
		AT	81	(47.6)	140	(48.4)	
		AA	24	(14.1)	44	(15.2)	
	
	intron 1	CC	68	(40.0)	108	(37.4)	NS
		CT	86	(50.6)	145	(50.2)	
		TT	16	(9.4)	36	(12.4)	
	
	exon 4	TT	156	(91.8)	268	(92.7)	NS
		CT	14	(8.2)	21	(7.3)	
		CC	0	(0.0)	0	(0.0)	

*NFKBIA*	3'UTR	GG	53	(31.2)	101	(35.0)	NS
		AG	90	(52.9)	133	(46.0)	
		AA	27	(15.9)	55	(19.0)	
	
	promoter -297	CC	107	(62.9)	213	(73.7)	p = 0.01*
		CT	54	(31.8)	72	(24.9)	p_c _= 0.18
		TT	9	(5.3)	4	(1.4)	OR = 1.65 (1.10-2.48)
	
	promoter -826	CC	101	(59.4)	205	(70.9)	p = 0.01*
		CT	59	(34.7)	80	(27.7)	p_c _= 0.18
		TT	10	(5.9)	4	(1.4)	OR = 1.67 (1.12-2.48)

* p value was calculated by a by chi-square (χ^2^) test using a 2 × 2 table to compare the frequency of genotypes possessing the T allele *vs*. CC homozygotes and corrected (p_c_) for the number of tests performed.

N - number of patients available for analysis.

NS - non significant.

**Table 5:** Frequencies of *NFKBIA *promoter haplotypes in subgroups of patients with Graves' disease stratified by clinical activity of thyroid associated ophtalmopathy assessed according to the NOSPECS classification.

**Gene**	**polymorphism 1**	**polymorphism 2**	**Ophtalmopathy**	
			
			**NOSPECS ≥ III**	**NOSPECS ≤ II**
*NFKBIA*	-297 C/T	-826 C/T	N = 170 (%)	N = 289 (%)

haplotypes	C	C	126 (74.0)	238 (82.3)
	T	T	31 (18.4)	33 (11.3)
	T	C	8 (4.8)	11 (3.9)
	C	T	5 (2.8)	7 (2.5)

Haploview software was used to construct haplotypes from population genotype data and to assess possible difference in their distribution. The distribution of *NFKBIA *promoter haplotypes differed significantly between subgroups of GD subjects stratified by the presence of clinically evident ophthalmopathy (p = 0.003).

No other associations between the studied *IKBL *and *NFKBIA *polymorphisms and the clinical phenotype of GD were observed (data not shown).

### Interaction studies with known susceptibility loci

Finally we analyzed the distribution of *IKBL *and *NFKBIA *alleles in subgroups of GD patients stratified by genetic markers: HLA DRB1*03, *CTLA4 *49G and *PTPN 22 *1858T alleles [see Additional file 2]. The frequency of the *IKBL *-62A allele was significantly greater in HLA DRB1*03 carriers compared to the rest of GD subjects (57.9% *vs. *25.7%, p < 10^-4^, p_c _< 10^-4^, OR = 14.32 [95%CI: 7.02-29.22], SP = 100%). For the *IKBL *exon 4 T/C SNP, the T allele was more frequent in HLA DRB1*03 carriers (96.1% *vs. *89.9%, p = 0.047, p_c _= 0.846; OR = 2.73 [95%CI: 0.98-7.60], SP = 52.2%). Moreover, we observed a higher frequency of the *IKBL *-62A allele in *CTLA4 *49G positive patients (65.4% *vs. *53.7%, p = 0.02, p_c _= 0.36; OR = 1.62 [95%CI: 1.09-2.44], SP = 65.7%).

## Discussion

The present study investigated a role of selected polymorphisms within genes encoding IκB: *IKBL *(encoding IκBL) and *NFKBIA *(encoding IκBα) in development of Grave's disease (GD) in the Polish population.

The first observation made in this study is a possible association of the two SNPs in the promoter (-62 T/A) and in the first intron (C/T) of the *IKBL *with susceptibility to GD. The *IKBL *gene is located on the telomeric end of the central MHC (major histocompatibility complex) on chromosome 6. Since it was initially described by Allock *et al. *[21], the -62 T/A SNP was found to disrupt an E-box binding element in the *IKBL *promoter - a sequence that can also be found in promoter and enhancer regions of the wide-variety of B and T cell lineage specific genes. Moreover, independent functional studies reported an association of the -62A allele with a decrease of the *IKBL *promoter activity that can result in disinhibition of the NF-κB mediated inflammatory response [22,23]. In our dataset we observed a higher frequency of the -62A allele in patients with GD compared to the healthy subjects. In Australians, this allele constituted a part of the diabetogenic haplotype [7,23]. On contrary, in the Japanese population an association between the -62T allele and development of rheumatoid arthritis was reported [6]. However, subsequent replication case-control and family-based studies failed to confirm this association in Caucasians, suggesting that genetic susceptibility linked to *IKBL *may vary in different ethnic groups [24,25].

While possible consequences of the -62 T/A SNP are examined, the functional importance of the C/T substitution in the first *IKBL *intron remains unknown. In the present study we observed an association of the intron1 T allele with development of GD in Polish population. In the previous report the T allele was linked to the resistance to rheumatoid arthritis in Japanese [6] and to our knowledge, this SNP has not been studied in other autoimmune diseases. In general, intronic polymorphisms may act as markers linked to the functional, causative variants [26]. In the studied population we observed a strong linkage disequilibrium between the promoter -62 T/A and intron 1 C/T SNPs, and therefore we suppose that the observed association may be secondary to the linkage with the -62A allele. Moreover, a preliminary interaction analysis with the established GD susceptibility markers revealed a possible interaction between the studied *IKBL *variants and a HLADRB1*03 alleles - known as probably the most significant genetic predisposing factor to GD in Caucasians. However genes located within the MHC complex are in a strong linkage disequilibrium and certain alleles may occur in combination at a greater frequency than would be predicted by random association. This phenomenon hinders the distinction between genes primarily involved in conferring susceptibility to a disease and markers for the effect of a nearby gene [27]. Therefore, a further study involving HLA-DR genotyping of the Polish controls, as well as adequately powered replication studies in other Caucasian populations are required to determine the independent role of *IKBL *SNPs and HLADRB1*03 alleles in pathogenesis of Graves' disease. The possible interaction between *IKBL *SNPs and CTLA-4 49A/G polymorphism must be judged very cautiously (statistical power < 80% and pc > 0.05).

The second IκB gene analyzed in this study was *NFKBIA*. In previous reports two promoter polymorphisms within this gene (-826 C/T and -297 C/T substitutions) were associated with prevalence of rheumatoid arthritis and systemic lupus erythematosus in the Taiwanese population [9,10]. *In silico *analysis revealed that the -297 C/T SNP is situated close to the sites of NF-κB binding in the *NFKBIA *promoter. In turn, the -826 C/T substitution disrupts a putative binding site of transcription factor GATA-2 [9,10]. In our dataset, the comparison of genotype and allele frequencies of these SNPs revealed no differences between GD patients and healthy subjects. However, when correlated with phenotype, the -297T and -826T alleles (alone, as well as in a haplotype) were found to be associated with presence of clinically evident ophthalmophaty. Interestingly, these alleles have been previously associated with development of chronic inflammatory diseases in Caucasians [28]. It is reported that pharmacological inhibition of the NF-κB pathway in patients with active ophthalmophaty results in the suppression of inflammation and in the decreased glycosaminoglycans production by orbital fibroblasts [29]. Therefore it can be assumed that allelic differences in the *NFKBIA *promoter that affect IκBα expression may influence regulation of the inflammatory response in the orbit tissue. However the genotype-phenotype correlation should be treated as preliminary since the analysis was underpowered and p_c _> 0.05. Future replication studies are required to confirm our observation.

## Conclusion

In conclusion, SNPs in genes encoding IκBL and IκBα may contribute to the development or clinical phenotype of GD. However, since it is not possible to conclude if these loci represent primary etiological variants, further replication and functional studies are required to evaluate a role of IκB polymorphisms in development of thyroid autoimmunity.

## Competing interests

There is no conflict of interest that could be perceived as prejudicing the impartiality of the research reported.

## Authors' contributions

AK carried out the molecular genetic studies, performed the statistical evaluation and drafted the manuscript, PM participated in acquisition of data, EBA and JN participated in design and coordination of the study, TB conceived of the study, participated in its design and coordination and revised the manuscript. All authors read and approved the manuscript.

## Supplementary Material

Additional file 1 **PCR-RFLP conditions used for the analysis of the selected polymorphisms in the *IKBL *and *NFKBIA *genes**. The data provided describe experimental conditions used for the PCR-RFLP analysis. Click here for file

Additional file 2 **Distribution of *IKBL *genotypes in subgroups of patients with GD stratified by genetic parameters**. The data provided represents the statistical analysis of the *IKBL *genotypes distribution in subgroups of patients with GD stratified by genetic parameters including: HLA DRB1*03, *CTLA4 *49G and *PTPN 22 *1858T alleles. Click here for file

## References

[B1] O'Sullivan B, Thompson A, Thomas R (2007). NF-kappa B as a therapeutic target in autoimmune disease. Expert Opin Ther Targets.

[B2] Hayden MS, West AP, Ghosh S (2006). NF-kappaB and the immune response. Oncogene.

[B3] Kumar A, Takada Y, Boriek AM, Aggarwal BB (2004). Nuclear factor-kappaB: its role in health and disease. J Mol Med.

[B4] Kurylowicz A, Hiromatsu Y, Jurecka-Lubieniecka B, Kula D, Kowalska M, Ichimura M, Koga H, Kaku H, Bar-Andziak E, Nauman J, Jarzab B, Ploski R, Bednarczuk T (2007). Association of NFKB1 -94ins/del ATTG promoter polymorphism with susceptibility to and phenotype of Graves' disease. Genes Immun.

[B5] Miterski B, Böhringer S, Klein W, Sindern E, Haupts M, Schimrigk S, Epplen JT (2002). Inhibitors in the NFkappaB cascade comprise prime candidate genes predisposing to multiple sclerosis, especially in selected combinations. Genes Immun.

[B6] Okamoto K, Makino S, Yoshikawa Y, Takaki A, Nagatsuka Y, Ota M, Tamiya G, Kimura A, Bahram S, Inoko H (2003). Identification of I kappa BL as the second major histocompatibility complex-linked susceptibility locus for rheumatoid arthritis. Am J Hum Genet.

[B7] Price P, Cheong KY, Boodhoo A, Witt CS, McCann V, Christiansen FT, Allcock RJ (2001). Can MHC class II genes mediate resistance to type 1 diabetes?. Immunol Cell Biol.

[B8] Lin CH, Cho CL, Tsai WC, Ou TT, Wu CC, Yen JH, Liu HW (2006). Inhibitors of kB-like gene polymorphisms in rheumatoid arthritis. Immunol Lett.

[B9] Lin CH, Ou TT, Wu CC, Tsai WC, Liu HW, Yen JH (2007). IkappaBalpha promoter polymorphisms in patients with rheumatoid arthritis. Int J Immunogenet.

[B10] Lin CH, Wang SC, Ou TT, Li RN, Tsai WC, Liu HW, Yen JH (2008). I kappa B alpha promoter polymorphisms in patients with systemic lupus erythematosus. J Clin Immunol.

[B11] Ou TT, Lin CH, Lin YC, Li RN, Tsai WC, Liu HW, Yen JH (2008). IkappaBalpha promoter polymorphisms in patients with primary Sjögren's syndrome. J Clin Immunol.

[B12] Klein W, Tromm A, Folwaczny C, Hagedorn M, Duerig N, Epplen JT, Schmiegel WH, Griga T (2004). A polymorphism of the NFKBIA gene is associated with Crohn's disease patients lacking a predisposing allele of the CARD15 gene. Int J Colorectal Dis.

[B13] Bednarczuk T, Hiromatsu Y, Seki N, Płoski R, Fukutani T, Kuryłowicz A, Jazdzewski K, Chojnowski K, Itoh K, Nauman J (2004). Association of tumor necrosis factor and human leukocyte antigen DRB1 alleles with Graves' ophthalmopathy. Hum Immunol.

[B14] Miller SA, Dykes DD, Polesky HF (1988). A simple salting out procedure for extracting DNA from human nucleated cells. Nucleic Acids Res.

[B15] Bednarczuk T, Hiromatsu Y, Fukutani T, Jazdzewski K, Miskiewicz P, Osikowska M, Nauman J (2003). Association of cytotoxic T-lymphocyte-associated antigen-4 (CTLA-4) gene polymorphism and non-genetic factors with Graves' ophthalmopathy in European and Japanese populations. Eur J Endocrinol.

[B16] Skórka A, Bednarczuk T, Bar-Andziak E, Nauman J, Ploski R (2005). Lymphoid tyrosine phosphatase (PTPN22/LYP) variant and Graves' disease in a Polish population: association and gene dose-dependent correlation with age of onset. Clin Endocrinol (Oxf).

[B17] Woolf B (1955). On estimating the relation between blood group and disease. Ann Hum Genet.

[B18] Barrett JC, Fry B, Maller J, Daly MJ (2005). Haploview: analysis and visualization of LD and haplotype maps. Bioinformatics.

[B19] Gabriel SB, Schaffner SF, Nguyen H, Moore JM, Roy J, Blumenstiel B, Higgins J, DeFelice M, Lochner A, Faggart M, Liu-Cordero SN, Rotimi C, Adeyemo A, Cooper R, Ward R, Lander ES, Daly MJ, Altshuler D (2002). The structure of haplotype blocks in the human genome. Science.

[B20] Ardlie KG, Kruglyak L, Seielstad M (2002). Patterns of linkage disequilibrium in the human genome. Nat Rev Genet.

[B21] Allcock RJ, Baluchova K, Cheong KY, Price P (2001). Haplotypic single nucleotide polymorphisms in the central MHC gene IKBL, a potential regulator of NF-kappaB function. Immunogenetics.

[B22] Ozaki K, Ohnishi Y, Iida A, Sekine A, Yamada R, Tsunoda T, Sato H, Sato H, Hori M, Nakamura Y, Tanaka T (2002). Functional SNPs in the lymphotoxin-alpha gene that are associated with susceptibility to myocardial infarction. Nat Genet.

[B23] Boodhoo A, Wong AM, Williamson D, Voon D, Lee S, Allcock RJ, Price P (2004). A promoter polymorphism in the central MHC gene, IKBL, influences the binding of transcription factors USF1 and E47 on disease-associated haplotypes. Gene Expr.

[B24] Kilding R, Iles MM, Timms JM, Worthington J, Wilson AG (2004). Additional genetic susceptibility for rheumatoid arthritis telomeric of the DRB1 locus. Arthritis Rheum.

[B25] Collado L, Rueda B, Cáliz R, Torres B, García A, Nuñez-Roldan A, González-Escribano MF, Martin J (2004). Lack of association between the I kappa BL promoter polymorphism and rheumatoid arthritis. Arthritis Rheum.

[B26] Brand OJ, Barrett JC, Simmonds MJ, Newby PR, McCabe CJ, Bruce CK, Kysela B, Carr-Smith JD, Brix T, Hunt PJ, Wiersinga WM, Hegedüs L, Connell J, Wass JA, Franklyn JA, Weetman AP, Heward JM, Gough SC (2009). Association of the thyroid stimulating hormone receptor gene (TSHR) with Graves' disease. Hum Mol Genet.

[B27] Burton PR, Clayton DG, Cardon LR, Craddock N, Deloukas P, Duncanson A, Kwiatkowski DP, McCarthy MI, Ouwehand WH, Samani NJ, Todd JA, Donnelly P, Barrett JC, Davison D, Easton D, Evans DM, Leung HT, Marchini JL, Morris AP, Spencer CC, Tobin MD, Attwood AP, Boorman JP, Cant B, Everson U, Hussey JM, Jolley JD, Knight AS, Wellcome Trust Case Control Consortium; Australo-Anglo-American Spondylitis Consortium (TASC) (2007). Association scan of 14,500 nonsynonymous SNPs in four diseases identifies autoimmunity variants. Nat Genet.

[B28] Abdallah A, Sato H, Grutters JC, Veeraraghavan S, Lympany PA, Ruven HJ, Bosch JM van den, Wells AU, du Bois RM, Welsh KI (2003). Inhibitor kappa B-alpha (IkappaB-alpha) promoter polymorphisms in UK and Dutch sarcoidosis. Genes Immun.

[B29] Cao HJ, Smith TJ (1999). Leukoregulin upregulation of prostaglandin endoperoxide H synthase-2 expression in human orbital fibroblasts. Am J Physiol.

