# Supplementary material for: Association of polymorphism in genes encoding κB inhibitors (IκB) with susceptibility to and phenotype of Graves' disease: a case-control study
Source: Thyroid Res. 2009 Nov 3;2:10. doi: 10.1186/1756-6614-2-10 (PMC2777844; doi:10.1186/1756-6614-2-10)
Supplement: Additional file 1 — PCR-RFLP conditions used for the analysis of the selected polymorphisms in the IKBL and NFKBIA genes. The data provided describe experimental conditions used for the PCR-RFLP analysis. [file 1756-6614-2-10-S1.doc]

**Additional file 1**

**Title:** PCR-RFLP conditions used for the analysis of the selected polymorphisms in the *IKBL* and *NFKBIA* genes.

| Gene | Polymorphism | Accession No | Primers | T_m_  [˚C] | MgCl_2_  [mM] | PCR product | Restriction enzyme | Alleles |
| --- | --- | --- | --- | --- | --- | --- | --- | --- |
| *IKBL* | promoter -62 T/A | rs2071592 | F: 5’**-**CGGATGGGGAAAATTTTT-3’ | 55 | 2.0 | 301bp | *Pvu*II | A: 301bp |
|  |  |  | R: 5’-AGTTCACTTCCGTCCTCCAG-3’ |  |  |  |  | T: 281bp; 20bp |
|  | intron 1 C/T | rs2071591 | F: 5’**-**ACGACGAAAGCGACGTTCT-3’ | 48 | 2.0 | 221bp | *BstN*I | C: 202bp; 19bp |
|  |  |  | R: 5’-ATCTATCATCTGATAAAGGACCA-3’ |  |  |  |  | T: 221bp |
|  | exon 4 C/T | rs3130062 | F: 5’-TGAGTCCTTCTCAGCCTGGT-3’ | 59 | 1.5 | 315bp | *Hpy99*I | C: 225bp; 90bp |
|  |  |  | R: 5’-ACATCACCAAATCGCCAGA-3’ |  |  |  |  | T: 315bp |
| *NFKBIA* | 3’UTR G/A | rs696 | F: 5’-GGCTGAAAGAACATGGACTTG-3’ | 59 | 1.5 | 424bp | *Hae*III | G: 306bp; 118bp |
|  |  |  | R: 5’-GTACACCATTTACAGGAGGG-3’ |  |  |  |  | A: 424bp |
|  | promoter -297 T/C | rs2233409 | F: 5’-CCAGCCATCATTTCCACTCT-3’ | 59 | 1.5 | 300bp | *Hpy8*I | T: 168bp, 131bp |
|  |  |  | R: 5’-GAGAAACTCCCTGCGATGAG-3’ |  |  |  |  | C: 300bp |
|  | promoter -826 C/T | rs2233406 | F: 5’-AGGTCCAATCGCGGTTAAG-3’ | 53 | 1.5 | 196bp | *Bsl*I | C: 95bp; 84bp; 17bp |
|  |  |  | R: 5’GGTGGTGTGGATACCTTGCAATG-3’ |  |  |  |  | T: 112bp; 84bp |

T_m_ – melting temperature, F – forward, R – reverse, bp – base pairs, UTR – untranslated region, _ indicates a mismatch nucleotide
