# Supplementary material for: Association of polymorphism in genes encoding κB inhibitors (IκB) with susceptibility to and phenotype of Graves' disease: a case-control study
Source: Thyroid Res. 2009 Nov 3;2:10. doi: 10.1186/1756-6614-2-10 (PMC2777844; doi:10.1186/1756-6614-2-10)
Supplement: Additional file 2 — Distribution of IKBL genotypes in subgroups of patients with GD stratified by genetic parameters. The data provided represents the statistical analysis of the IKBL genotypes distribution in subgroups of patients with GD stratified by genetic parameters including: HLA DRB1*03, CTLA4 49G and PTPN 22 1858T alleles. [file 1756-6614-2-10-S2.doc]

**Additional file 2**

**Title:** Distribution of *IKBL* genotypes in subgroups of patients with GD stratified by genetic parameters.

| Polymorphism | Genotypes | *HLA DRB1*03 allele* | | p/p_c_ value** | *CTLA4* 49G *allele* | | p/p_c_ value** | *PTPN22* 1858T *allele* | | p/p_c_ value** |
| --- | --- | --- | --- | --- | --- | --- | --- | --- | --- | --- |
|  |  | present*  N=127(%) | absent  N=169(%) |  | present  N=347(%) | absent  N=134(%) |  | present  N=163(%) | absent  N=317(%) |  |
| *IKBL* | AA+AT | 117 (92.1) | 76 (45.0) | p< 10^-4^ | 227 (65.4) | 72 (53.7) | p=0.02 | 96 (58.9) | 203 (64.0) | NS |
| promoter -62 | TT | 10 (7.9) | 93 (55.0) | p_c_< 10^-4^ | 120 (34.6) | 62 (46.3) | p_c_=0.36 | 67 (41.1) | 67 (36.0) |  |
| *IKBL* | TT+CT | 119 (93.7) | 67 (39.6) | p< 10^-4^ | 221 (63.7) | 72 (53.7) | p=0.046 | 92 (56.4) | 201 (63.4) | NS |
| intron 1 | CC | 8 (6.3) | 102 (60.4) | p_c_< 10^-4^ | 126 (36.3) | 62 (46.3) | p_c_=828 | 71 (43.6) | 116 (36.6) |  |
| *IKBL* | TT | 122 (96.1) | 152 (89.9) | p=0.047 | 318 (91.6) | 123 (91.8) | NS | 148 (90.8) | 292 (92.1) | NS |
| exon 4 | CT | 5 (3.9) | 17 (10.1) | p_c_=0.846 | 29 (8.4) | 11 (8.2) |  | 15 (9.2) | 25 (7.9) |  |

N – number of patients available for analysis, NS – non significant.

*“present” = homozygotes possessing the investigated allele + heterozygotes.

** p values were calculated by chi-square (χ^2^) test using a 2x2 table to compare the frequency of alleles in subgroups of patients with Graves’ disease stratified by genetic parameters and corrected (p_c_) for the number of tests performed.
